# Prophylactic cranial irradiation in ES-SCLC: The ongoing debate from the past to the immunotherapy Era”

**DOI:** 10.1016/j.ctro.2026.101153

**Published:** 2026-03-21

**Authors:** Tala Najdi, Antoine Richa, Mia Chamieh, Toufic Zeidan, Fares Azoury, Carine Harmouche, Hampig Raphael Kourie

**Affiliations:** a Department of Hematology-Oncology, Hotel-Dieu de France University Hospital, Beirut, Lebanon; b Saint Joseph University, Beirut, Lebanon; c Department of Radiation Oncology, Hotel-Dieu de France University Hospital, Beirut, Lebanon; d Department of Pneumology, Hotel-Dieu de France University Hospital, Beirut, Lebanon; e Department of Medical Oncology, Institut Gustave Roussy, Villejuif, France

**Keywords:** extensive stage small cell lung cancer (ES-SCLC), prophylactic cranial irradiation (PCI), Immunotherapy, Radiotherapy, Brain metastases

## Abstract

SCLC (Small Cell Lung Cancer) is categorized into two stages: limited-stage (LS-SCLC), where the cancer is confined to a single radiation field, and extensive-stage, where the disease has spread beyond the chest, potentially including the brain. In managing extensive-stage SCLC (ES-SCLC), preventing brain metastases becomes a crucial goal, as these metastases are a major cause of mortality due to their severe complications. Prophylactic cranial irradiation (PCI) has been shown to reduce the incidence of brain metastases and, according to the pre-immunotherapy *meta*-analysis by Auperin et al., improve overall survival (OS). With the advent of chemoimmunotherapy in extensive-stage SCLC, the role of PCI is being re-evaluated, as immunotherapy may enhance systemic control, improve survival, and lower the risk of brain metastases, raising questions about the continued necessity of PCI. This review examines key studies that shaped prior guidelines and the evolving understanding of PCI’s effectiveness in the current treatment landscape. It evaluates both studies supporting and questioning PCI’s role, given the enhanced systemic control with immuno-chemotherapy, while also addressing the risks and long-term complications of PCI, including cognitive impacts and quality of life concerns. Additionally, the review explores alternative strategies, such as MRI surveillance, and identifies which patient populations are most likely to benefit from PCI and in which patients PCI can be omitted.

## Introduction

Small cell lung cancer, a highly aggressive neuroendocrine carcinoma, accounts for approximately 13% of all lung cancers [1]. Extensive-stage small cell lung cancer (ES-SCLC) refers to disease that has spread beyond the hemithorax and is often associated with poor prognosis. As many as 80% of patients with SCLC may develop brain metastases during the course of their disease, including occult brain metastases [2]. Brain metastases can significantly impact the patient’s quality of life and negatively impact overall survival (OS). Prophylactic cranial irradiation (PCI) can prevent brain metastases formation and seeding thus reducing the risk of micro metastatic disease in the brain and ultimately improving survival. PCI is a standard of care in patients with limited stage SCLC (LS-SCLC) with partial or complete response to systemic therapy [3].

In ES-SCLC, the role of PCI has become increasingly debated. The introduction of immunotherapy, which enhances systemic control, has allowed some patients to achieve prolonged survival and excellent disease management. While pre-immunotherapy studies fueled controversy over PCI’s impact on OS and the development of brain metastases, the advent of chemoimmunotherapy further questions its therapeutic benefit in this setting [4]. In this review, we examine the available literature to provide a clearer understanding of current practices and challenges, aiming to help clinicians make informed, personalized decisions about whether to offer PCI to patients with ES-SCLC. Moreover, PCI is associated with several potential complications and side effects—both acute and long-term—that can affect quality of life and cognitive function. Therefore, it is crucial to ensure that the potential benefits outweigh these risks. This review also explores strategies to mitigate PCI-related toxicity, such as dose optimization and hippocampal-sparing techniques.

## Methods

We conducted a narrative review of key clinical trials that have shaped the current understanding of prophylactic cranial irradiation (PCI) in extensive-stage small cell lung cancer (ES-SCLC). This included a detailed examination of the National Comprehensive Cancer Network (NCCN), European and American clinical guidelines to assess the evolution of treatment recommendations. Studies were explored in UpToDate and MEDLINE using the keywords “PCI,” “ES-SCLC,” and “immunotherapy.” We reviewed relevant studies supporting the use of PCI as well as those that have raised concerns or questioned its benefit after the emergence of immunotherapy in combination with chemotherapy. Based on these findings, we engaged in a discussion to critically analyze the current treatment landscape.

## Results

### PCI in ES-SCLC prior to immunotherapy era

In the landmark *meta*-analysis by Aupérin et al. (1999), PCI improved both overall (20.7% in the treatment group vs 15.3% in the control group) and disease-free survival (HR, 0.73; 95% CI, 0.65–0.86; P < 0.001) in patients with SCLC in complete remission, of whom 15% had ES [5].

PCI has long been associated with a reduction in brain metastases. Before immunotherapy became part of standard treatment, Slotman et al. (2007) conducted a study in 286 patients who had responded to chemotherapy, randomizing them to receive prophylactic cranial irradiation or no further therapy. It is important to note that brain imaging was not mandatory in the initial staging, but it was performed when any predefined key symptom suggestive of brain metastases was present. The two groups (each with 143 patients) were well balanced regarding baseline characteristics. Patients who received PCI had a lower risk of symptomatic brain metastasis: the cumulative risk of brain metastases within one year was 14.6% in the irradiation group, compared to 40.4% in the control group with a HR for the irradiation group of 0.27 (95% CI, 0.16 to 0.44). In this trial, only 59% of patients in the observation arm who developed brain metastases underwent salvage radiation. Additionally, this resulted in a significant improvement in OS for patients in the irradiation group (p = 0.03), with a median survival of 6.7 months, as compared with 5.4 months in the control group with a HR of 0.68 (95% CI, 0.52 to 0.88). There was no significant effect on neurocognitive function or overall impairment between the two arms. As a result, PCI has become the standard treatment for SCLC patients who show a positive response to initial therapy [6].

In contrast, Takahashi’s randomized trial recruited patients with ES-SCLC who had completed two or more cycles of platinum-based doublet chemotherapy. In this study, the absence of brain metastases, confirmed by gadolinium-enhanced MRI, was required for enrollment. Patients in the control group underwent active surveillance with MRI every three months, independent of symptoms, whereas those in the treatment arm received PCI at a total dose of 25 Gy in 10 fractions. At 12 months, the cumulative incidence of brain metastases was 32.9% in the PCI group compared to 59.0% in the observation arm, with the difference being statistically significant (p < 0.0001). However, neither median overall survival (mOS) (11.6 versus(vs) 13.1 months, p = 0.09) nor progression-free survival (PFS)(2.3 vs 2.4, p = 0.75) showed statistically significant differences [7]. The study used the mini-mental state examination (MMSE) to assess cognitive function before, 12 months after and 24 months after randomization. The scores between the PCI group and observation group did not show any significant difference at any time point (12 months p = 0.71, 24 months p = 0.52). The conflicting results of these two studies are presented in Table 1 below.

**Table 1:** Comparison of PCI in ES-SCLC: Results from the Slotman and Takahashi Studies.

Trial	Author	N	Brain staging and surveillance requirements		Arms of the study	Endpoints
			Before randomization	Surveillance		
EORTC trial (2007) [6]	Slotman B et.al	286	No	Symptomatic neurologic	PCI after chemo	1-year risk incidence of brain met: % (p < 0.001) PCI: 14.6% No PCI: 40.4% mOS (p = 0.003) PCI: 6.7 mo No PCI: 5.4 mo DFS (p = 0.02) PCI: 14.7 weeks No PCI: 12.0 weeks
					No irradiation after chemo	
Japanese trial (2017) [7]	Takahashi et.al	224	Yes	Yes	PCI after chemo	mOS (p = 0.09) PCI: 11.6 mo No PCI: 13.1 mo PFS (p = 0.75) PCI: 2.4 mo No PCI: 2.3 mo Incidence of brain met at 1 year (p < 0.0001) PCI: 32.9% No PCI: 59.0%
					Observation after chemo	

Abbreviations: EORTC, European Organization for Research and Treatment of Cancer; mOS, median overall survival; DFS: disease free survival vs: versus; PFS, progression free survival; mo: months; PCI, prophylactic cranial irradiation; met, metastases, chemo, chemotherapy.

The CALGB 30504 phase II trial did not address the efficacy of PCI, but rather evaluated maintenance sunitinib vs placebo in ES-SCLC patients responding to platinum-based therapy. Maintenance sunitinib improved PFS (3.7 vs. 2.1 months, HR 1.62, p = 0.02) without a significant OS benefit (9.0 vs. 6.9 months, HR 1.28, p = 0.16). PCI was permitted in both study arms, without influencing progression-free survival[8].

Most of the studies conducted after the EORTC study suggest that PCI does not provide a benefit in OS, although it may impact the incidence of brain metastasis and PFS. A 2021 study for example, in the MRI era, examined the effectiveness of PCI for ES-SCLC through MRI screening and found that nearly 16% of patients had undetected brain metastases near the end of chemotherapy. In this retrospective cohort, PCI significantly reduced brain metastases over one year, though it did not show a survival benefit [9]. Consistent with these findings, a 2024 re-examination of PCI confirmed that PCI is associated with a survival benefit overall; however, this benefit was not evident in studies that radiographically excluded brain metastases using MRI (HR 0.74; 95% CI, 0.52–1.05; p = 0.08; 9 studies; 1,384 patients).This implies that patient selection may play a role in these findings [10].

Moreover, PCI is associated with several toxicities, primarily impacting cognitive function. Research indicates that PCI can lead to a decline in both tested and self-reported cognitive abilities. A pooled secondary analysis of RTOG trials 0212 and 0214 demonstrated that PCI recipients exhibited a threefold increased risk of cognitive decline, particularly affecting memory and delayed recall, as measured by the Hopkins Verbal Learning Test (HVLT). Furthermore, self-reported cognitive decline, assessed via the EORTC QLQ-C30 questionnaire, was significantly higher in the PCI group compared to those who did not undergo irradiation [11]. Although these two trials were conducted in limited-stage SCLC and NSCLC (non small cell lung cancer), respectively, the neurocognitive impact of cranial irradiation is likely similar in the setting of ES disease.

Following the study of Takahashi et.al, Gjyishi et.al conducted in 2019 a nationwide survey study to assess changes in the practice of radiation oncologists (ROs) regarding PCI in ES-SCLC. In fact, routine recommendations decreased from 72% to 44%. Additionally, 82% of clinicians expressed willingness to enroll patients in a trial of MRI surveillance with or without PCI for both limited and ES-SCLC [12].

Considering these conflicting results, the National Comprehensive Cancer Network (NCCN) established, in 2018, equipoise between MRI surveillance and PCI in ES-SCLC (level 2A). Therefore, the NCCN guidelines now consider PCI as 'optional' for extensive-stage SCLC and advises MRI surveillance for all patients, irrespective of PCI treatment. The surveillance protocol includes an MRI every 3 to 4 months for one year, then every 6 months for 2 years [13].

The European guidelines still recommend PCI for patients under 75 years old, with a performance status (PS) from 0 to 2 and an MRI is not required beforehand [14]. The American Society for Radiation Oncology (ASTRO) recommends a consultation with a radiation oncologist for patients with ES-SCLC who respond to chemotherapy for evaluation of PCI [15].

These guidelines and recommendations are summarized in Table 2 below.

**Table 2:** Main Guidelines and Recommendations Findings.

**Guideline/Trial**	**Recommendation/Findings**	**Details/Notes**
**NCCN Guidelines (2018)**[13]	PCI is 'optional' for ES-SCLC	MRI surveillance is recommended every 3–4 months for 1 year, then every 6 months for 2 years. Level 2A recommendation.
**European Guidelines**[14]	PCI is recommended for patients under 75 years old with a PS of 0–2.	MRI was not mandatory before PCI.
**ASTRO Guidelines**[15]	Consultation with a radiation oncologist is recommended for patients who respond to chemotherapy to decide for PCI	Emphasizes personalized treatment based on patient characteristics, rather than on imaging results like MRI.
**Takahashi et al. (Japanese study)**[7]	PCI led to a decrease in brain met incidence but not an improvement in overall survival	Neurotoxicity and cognitive decline were higher with PCI

Abbreviations: PCI, prophylactic cranial irradiation; MRI, magnetic resonance imaging; OS, overall survival; PS, performance status; met, metastases.

Both American and European guidelines recommend PCI at 25 Gy in 10 fractions or 20 Gy in 5 fractions, based mainly on studies in LS-SCLC. Two trials tested higher doses (36 Gy). In the study by Le Péchoux et al., higher-dose PCI did not reduce brain metastases and was associated with worse 2-year OS (37% vs. 42% with 25 Gy; HR 1.20, p = 0.05) [16]. Similarly, RTOG 0212 showed no survival benefit but increased chronic neurotoxicity, reinforcing 25 Gy as the standard dose for patients in complete remission after chemotherapy and thoracic radiotherapy [17].

### PCI with the emergence of immunotherapy

Since the ESMO 2021 recommendations, chemo-immunotherapy has become the new standard of care after more than 25 years with no improvements in the context on ES-SCLC [14]. A retrospective study examining the decline in PCI rates following the introduction of immunotherapy found that the overall rate of documented PCI for patients receiving either first-line immunotherapy or first-line chemotherapy decreased from 14.7% in 2013 to 7.0% in 2018–2019 (p < 0.001) during the study period [18].

The combination of immunotherapy and chemotherapy using atezolizumab or durvalumab in concomitant and maintenance treatment has been implemented following the IMpower133 and CASPIAN trials. In the IMpower133 trial, EP plus atezolizumab improved mOS to 12.3 months vs 10.3 months with EP plus placebo (HR 0.70; p = 0.007)[19]. Patients with treated, asymptomatic brain metastases were eligible, while those with active or untreated CNS metastases were excluded. PCI was optional, with only 11% of patients receiving it in each arm. PCI improved median intracranial PFS in both arms: 20.2 vs 16.5 months with atezolizumab and 10.5 vs 9.8 months with placebo [20], [21].

In the Caspian Study, an overall improvement in survival was demonstrated in the Durvalumab plus EP with a mOS of 12.9 vs 10.5 months in the EP group (HR 0·75, p = 0.0032). In this study, asymptomatic untreated or treated and stable brain metastasis were permitted. Brain imaging was optional and not mandatory for patients with suspected brain metastases [22]. At baseline, brain metastases were present in 10.4% of patients in the durvalumab plus EP arm and 10.0% in the EP arm. PCI was allowed in the control group of chemotherapy alone not in the experimental group with immunotherapy due safety concern. The significant OS difference was seen regardless of brain metastasis, regardless of PCI. In patients without brain metastases, similar proportions in each arm developed new brain lesions (8.8% and 9.5%), with 8.3% of patients in the EP arm receiving PCI [2], [23].

Other studies incorporated immunotherapy in ES-SCLC such as pembrolizumab in Keynote 604 and nivolumab and ipilumab in CheckMate 451 but did not yield significant results.

In Keynote 604, patients were randomized 1:1 to pembrolizumab or placebo plus four cycles of standard-dose EP. The pembrolizumab arm had slightly more baseline brain metastases (14% vs. 10%). PCI was allowed after cycle 4 for responders, with 11.8% and 14.2% of patients receiving PCI in the pembrolizumab and placebo arms, respectively. Median OS was 10.8 months vs 9.7 months (HR 0.80; p = 0.0162), not meeting the prespecified significance threshold. In patients with brain metastases, hazard ratios for PFS and OS were > 1, indicating no benefit of pembrolizumab in this subgroup. The study did not report multivariate analyses or intracranial PFS outcomes for the PCI subgroup and in the context of first-line pembrolizumab plus EP, the role of PCI remains uncertain [24]. In CheckMate 451, maintenance therapy with nivolumab plus ipilimumab did not prolong OS compared to placebo. No significant OS difference was observed between PCI and non-PCI patients and no specific data of intracranial PFD differences between the groups was mentioned [25]. The main outcome of these studies is detailed in Table 3 below.

**Table 3:** Main studies investigating the use of immunotherapy and PCI in ES-SCLC.

Study (year)	N	Brain Met Allowed	Control Arm Experimental Arm	PCI allowed	Outcomes
IMPOWER 133, 2018 [19]	403	Yes asymptomatic, or treated Untreated brain met were excluded	Atezo + EP → Atezo	Yes (11%)	mOS (HR 0.70; p = 0.007) Atezo Arm: 12.3 mo Placebo Arm:10.3 mo **Median time to ICP** Atezo Arm: 20.2 with PCI vs 16.5mo if no PCI Placebo arm: 10.5mo with PCI vs 9.8 mo if no PCI
			Placebo + EP → Placebo	Yes (11%)	
CASPIAN, 2018 2022 (update)[22]	537 (805 total)	Yes, asymptomatic and/or treated	Durva + EP → Durva	Yes (8.3%)	mOS (HR 0.75; p = 0.0032) Durva: 12.9mo Placebo: 10.5 mo **Incidence of new brain met** Durva Arm: 8.8% Placebo Arm: 9.5%
			Placebo + EP → Placebo	No	
Keynote 604, 2020 [24]	453	Yes, asymptomatic and/or treated	Pembro + EP → Pembro	Yes (14.2%)	mOS (HR 0.8; p = 0.0162) Pembro Arm: 10.8mo Placebo Arm: 9.7mo **No specific intracranial PFS outcomes**
			Placebo + EP → Placebo	Yes (11.8%)	
CheckMate 451, 2021 [25]	834	Yes, asymptomatic and/or treated	EP → Nivo + Ipi	Yes 22.9%	mOS Nivo + Ipi arm: 9.2 mo (HR 0.92) Nivo arm: 10.4mo (HR 0.84) Placebo arm: 9.6 mo **No significant OS difference was seen between the groups regardless of PCI**
			EP → Nivo	Yes 22.2%	
			EP → Placebo		

Abbreviations: N, population number; mOS, median overall survival; OS, overall survival; HR, Hazard ration; Met, metastasis; PCI, prophylactic irradiation; mo, months; EP, platine + etoposide; Atezo, Atezolizuma; Durva, Durvalumab; Nivo + Ipi, Nivolumab + Ipilumab;

Another immunotherapy regimen was studied in a multicenter, double-blind phase 3 clinical trial conducted in the People's Republic of China. In April 2020, with a median follow-up of approximately 14.4 months, Tislelizumab plus chemotherapy exhibited a statistically significant OS benefit vs placebo plus chemotherapy (15.5 vs 13.5 months, HR = 0.75, p = 0.0040) without the addition of PCI. PCI was associated with a trend toward improved mPFS (2.9 vs. 2.2 months, HR = 0.69), but not mOS [26].

Diving deeper in the ongoing debate of the benefit of PCI in ES-SCLC, a single-center retrospective study evaluated the impact of PCI in 56 ES-SCLC patients without brain metastases (25 received PCI, 31 did not). Eighteen patients received immunotherapy, mostly in the non-PCI group. With a median follow-up of 16 months, mOS was 11.7 months with PCI vs 20.3 months without PCI (p = 0.412), and brain metastasis-free survival was 13.4 vs 10.7 months (p = 0.336), with no significant differences. Recursive partitioning analysis identified performance status, immunotherapy, and age, not PCI, as the main predictors of OS. [27]. In another single institution series of 163 patients with ES-SCLC without intracranial metastasis treated with or without immunotherapy, the addition of PCI did not improve neither OS nor PFS [28].

Given the conflicting results and the absence of randomized trials assessing the true efficacy of PCI, several ongoing prospective studies, listed in Table 4, are attempting to address this controversial issue.

**Table 4:** Ongoing clinical trials on prophylactic cranial irradiation in ES-SCLC.

Trial	Phase	Date	Enrolled	Control	Experimental arm	Status	Primary outcomes
MAVERICK (SWOG 1827) [29]	3	2020– (2027)	(668)	MRI active surveillance	PCI + MRI surveillance	Recruiting	OS
PRIMALung (NCT04790253) [29]	3	2023– (2028)	(600)	MRI active surveillance	PCI + MRI surveillance	Recruiting	OS
NCT04947774 [31]	NA	2020–2022	100	Observation	PCI	Unknown status	PFS in the brain
NCT04535739 [32]	3	2019–2022	414	Thoracic radiotherapy	PCI + thoracic radiotherapy	Unknown status	OS
NRG-CC003 (NCT02635009) [44][44]	2/3	2015–2027	418	PCI using 3DCRT	PCI with HA using IMRT	Recruiting	Deterioration in HVLT-R Intracranial relapse

Abbreviations: MRI, Magnetic Resonance Imaging; PCI, prophylactic cranial irradiation; 3DCRT, three-dimensional conformal radiation therapy; IMRT, Intensity-Modulated Radiation Therapy; HA, hippocampal avoidance; HVLT-R, Hopkins Verbal Learning Test – Revised.

The MAVERICK (SWOG1827) (MRI brain surveillance alone versus MRI surveillance and prophylactic cranial irradiation) is a randomized phase III trial testing the de-escalating approach of surveillance vs PCI. S1827 was designed to be highly inclusive of the diverse and evolving approaches to care in SCLC, such as hippocampal avoidance, memantine, immunotherapy, salvage WBRT, and SRS. Concomitant or adjuvant immunotherapy is allowed. It is important to note that in patients receiving chemotherapy plus immunotherapy followed by adjuvant immunotherapy, enrollment should occur after the chemotherapy phase [29]. The primary objective is to demonstrate that OS with brain MRI surveillance alone is non-inferior to that with the addition of PCI.

Similarly, the EORTC-1901 PRIMALung trial (NCT04790253), initiated in 2023, randomizes patients 1:1 to MRI surveillance with or without PCI (25  Gy in 10 fractions), also allowing immunotherapy [30]. This study aims to assess whether MRI alone provides non-inferior survival while preserving cognition, helping to clarify the role of PCI in SCLC and identify patients best suited for MRI monitoring.

Another two prospective Chinese trials are recruiting patients; NCT04947774 enrolled patients with ES-SCLC who responded to standard first-line therapy and aims to assess the safety and effectiveness of PCI, with PFS in the brain as the primary endpoint [31]. In contrast, NCT04535739 focuses on patients receiving PCI after chemotherapy and thoracic radiation therapy, with OS as the primary endpoint, a benefit recently supported by a retrospective real-world study showing that PCI was independently associated with improved OS in this population [32], [33]. While both studies are prospective and conducted in Chinese populations, the key difference lies in the timing of PCI: one after standard first-line treatment, the other following additional thoracic radiation. These studies will provide a clearer understanding of PCI, with the first trial (NCT04947774) incorporating immunotherapy as part of standard first-line treatment.

**Fig. 1:**
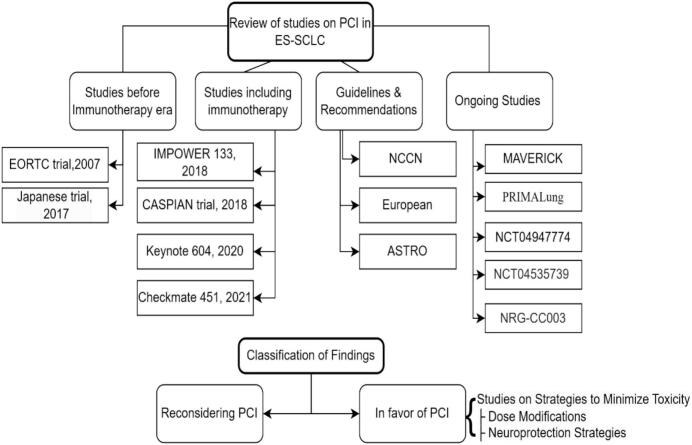
Review strategy. *Abbreviations: ES-SCLC, extensive-stage small cell lung cancer; NCCN: National Comprehensive Cancer Network; EORTC, European Organization for Research and Treatment of Cancer; ASTRO, American Society for Radiation Oncology.*

The flowchart (Fig. 1) below summarizes the review strategy adopted for searching and classifying findings.

## Discussion

After reviewing these studies, administering or not PCI in SCLC remains a challenge. Guidelines continue to be controversial, with NCCN advocating for replacing PCI with active surveillance, while European guidelines still recommend PCI for patients taking into consideration age and performance status (for patients under 75 years old with a PS of 0 to 2). An Italian national survey on behalf of the Italian association of radiotherapy and clinical oncology (AIRO) was conducted and respondents were more likely to perform PCI based on age and thoracic response (46%), while 30% considered only thoracic response, and 15% never offered PCI for ES-SCLC [34].

### In favor of PCI

Since 1999, Aupérin et al. demonstrated that PCI improves both OS and DFS in SCLC. The well-known EORTC study was initially considered one of the most important trials to demonstrate the benefit of PCI in SCLC in reducing ‘symptomatic brain metastases’, however, it has since faced criticism. In the EORTC trial, it’s important to note that brain imaging was not required before enrollment, so some patients with undiagnosed, asymptomatic brain metastases may have been included, potentially impacting the results. The benefit of radiation may have resulted from treating these hidden brain metastases, which were not identified due to the lack of imaging. A significant 41% of patients in the observation arm of the EORTC trial who developed symptomatic brain metastases did not undergo radiation. While the specific factors influencing decisions on salvage treatment remain unclear, it is likely that delayed detection of brain metastases in the trial negatively impacted performance status, reducing the proportion of patients eligible for salvage therapy and potentially leading to the difference in OS between the two arms. That’s why a substantial concern has emerged regarding the broader applicability of this study.

Although IMpower133 does not establish PCI as a standard of care in ES-SCLC, it supports its role in delaying the development of brain metastases. In the PCI group, the time to intracranial progression was longer compared to the non-PCI group, regardless of whether patients received atezolizumab or placebo. Additionally, a phase III trial exploring newer immunotherapies as the combination of Tislelizumab plus chemotherapy, PCI showed a trend toward improved mPFS.

Additionally, a *meta*-analysis of ES-SCLC reviewing 8 studies with 982 patients who received PCI and 4,509 who did not, showed that PCI improves 1-year OS and reduces the risk of brain metastasis [35].

### Reconsidering PCI

The Japanese study by Takahashi et al, raised skepticism about the results of the EORTC trial. In fact, the requirement for brain imaging before PCI may have contributed to the lack of difference in OS. If brain imaging is feasible, active surveillance could potentially replace PCI and its associated toxicities. Either way, both the EORTC and Japanese studies specifically evaluated PCI in the pre-immunotherapy era.

Another approach that may cast further doubt on the true benefit of PCI is the emergence of studies evaluating stereotactic radiosurgery (SRS) in the management of brain metastases in SCLC. While stereotactic radiotherapy is preferred for treating limited brain metastases from most histologies, WBRT has remained the standard of care for patients with SCLC because of a lack of prospective data supporting it. Bernhardt et al, suggested that SRS alone can be a viable alternative to WBRT for selected patients with SCLC brain metastases. While WBRT provides better CNS control, it does not improve OS and may lead to cognitive decline. The decision between SRS and WBRT should consider patient quality of life, the number of brain metastases, and individual clinical factors [36]. A recent prospective, multi-institutional study suggested that stereotactic approaches may offer an advantage over WBRT in SCLC patients with 1–10 brain metastases, demonstrating markedly lower rates of neurologic death (20 vs. 64) when patients are closely monitored after treatment [37]. Therefore, early detection by surveillance MRI and subsequent SRS treatment may compensate for the need of PCI.

After the addition of immunotherapy, the debate around the benefit of PCI in extensive disease has intensified. Even if IMpower 133 trial showed PCI’s potential in delaying brain metastasis appearance, the OS benefit was seen regardless of PCI. Similarly in the CASPIAN trial, the development of new brain metastases was similar in both groups, whether they received PCI or not, and the significant OS between the two arms was observed regardless of brain metastasis or PCI. Both the CASPIAN and IMpower 133 trials saw limited adoption of PCI, and the absence of proper controls restricts the understanding of its benefits. It is important to note that PCI was permitted in both arms in IMpower 133 but only in the placebo arm in the Caspian. In fact, by restricting PCI to placebo, researchers could assess whether immunotherapy itself offered better control over brain metastasis or if PCI was still necessary in the absence of immunotherapy.

The similar outcomes observed in studies including immunotherapy, even when PCI is omitted, may be due to the lack of PCI’s benefit or the introduction of immunotherapy, making it more difficult to draw definitive conclusions. Prospective evidence from ongoing trials. such as MAVERICK, PRIMALung, is needed to clarify PCI’s role.

### Minimizing PCI toxicities

Due to the conflicting clinical trials data, Kim et al. evaluated the cost-effectiveness of PCI vs MRI surveillance for ES-SCLC. Cost-effectiveness was assessed based on quality-adjusted life years (QALYs), with a willingness to pay (WTP) threshold of $100,000 per QALY gained. The study found that PCI was not cost-effective compared to MRI surveillance. The limited survival benefit (2.5 months) and neurocognitive decline from PCI contributed to its lower cost-effectiveness. Consequently, they concluded that MRI surveillance with early salvage therapy remains a viable strategy**,** and the role of PCI should be reassessed [38].We know that PCI affects neurocognitive end points as revealed by RTOG 0212 and 0214 reporting a threefold increase in patient-reported cognitive decline at 6 and 12 months with PCI especially in older age [17], [39].

hippocampal-avoidance PCI (HA-PCI)**.** The decline in delayed free recall (DFR) on the Hopkins Verbal Learning Test–Revised (HVLT-R) is used as a primary endpoint to assess cognitive function after PCI in many trials. Back in March 2011, the RTOG phase III study demonstrated that hippocampal neural stem-cell injury during WBRT may contribute to memory decline. By avoiding the hippocampus during irradiation, the study showed a smaller decline in HVLT-R delayed recall from baseline to 4 months, estimated at 7.0% (95% CI, –4.7% to 18.7%) [40]. This finding was later reaffirmed; The PREMER randomized Phase III trial (GICOR-GOECP-SEOR) enrolling 150 patients with SCLC showed that HA-PCI significantly preserves cognitive function compared with standard PCI, without affecting OS. At 3 months, the DFR was lower in the HA-PCI arm (5.8%) vs the PCI arm (23.5%; 95% CI, 1.57–15.86; P = 0.003)[41]. At the same time, the dutch prospective trial (NCT01780675) using the HVLT-R at 4 months did not show that hippocampal sparing lowered the cognitive decline with similar ≥ 5-point drops occurring in 29% of PCI patients and 28% of HA-PCI patients (p = 1.000) [42]. Both trials showed no increase in brain metastases with HA-PCI, a finding of safety confirmed in a pooled analysis (sHR 1.03; 95% CI, 0.62–1.70; p = 0.91) [43]. This analysis highlighted the importance of MRI compliance and showed that patients who can maintain regular MRI and cognitive assessments may benefit most from HA-PCI. A larger trial, the NRG CC003, randomized 393 patients and later reported in 2025 that hippocampal avoidance during PCI reduce neurocognitive function failure and demonstrate a non-inferior 12-month intracranial recurrence risk (PCI 14.8% *v* HA-PCI 14.7%, *P* < 0.0001), with comparable survival (adjusted HR, 0.88 [95% CI, 0.67 to 1.14]; *P* = 0.33) and grade ≥ 3 toxicity (PCI 31.4% *v* HA-PCI 30.7%, *P* = 0.88) [44]. These trials are important because they demonstrate that hippocampal avoidance during PCI is worth considering, as it preserves brain control and OS and self-reported cognitive impairment, though further studies are needed to determine its consistent longitudinal cognitive benefit. In addition, the potential role of neuroprotective agents should be further investigated in future studies; Brown et.al demonstrated that adding memantine to WBRT delayed the onset of cognitive decline and his trial NRG-CC001 showed that HA-WBRT plus memantine preserved cognitive function better than WBRT plus memantine (adjusted hazard ratio, 0.74; 95% CI, 0.58 to 0.95; *P* = 0.02) [45], [46].

As noted above, key trials demonstrate that adding ICIs enhances systemic disease control and OS, emphasizing the importance of managing CNS diseases. If PCI is utilized, it should be done with minimized side effects, while MRI surveillance could serve as a more effective alternative, offering better neurocognitive protection and potentially being more cost-effective.

In the light of this discussion, for patients with ES-SCLC responding after immunotherapy, the decision to offer PCI depends on their overall condition and expected benefit. We could suggest that patients with good performance status (PS 0–1) may proceed to further evaluation, while fragile patients (PS ≥ 2, comorbidities, cognitive impairment, or advanced age) should forgo PCI in favor of MRI surveillance. PCI may be recommended for those with a prolonged life expectancy (>6–12 months) and low neurocognitive risk. For patients at high risk of cognitive decline, PCI may be omitted in favor of regular MRI monitoring per the NCCN guidelines with neurological assessments, and possible SRS can be advised depending on the number of brain metastases [36]. As highlighted in the *meta*-analysis re-examining PCI, systematic MRI screening may serve as an alternative to PCI in appropriately selected patients by enabling active surveillance while sparing others from unnecessary toxicities [10].

**Fig. 2:**
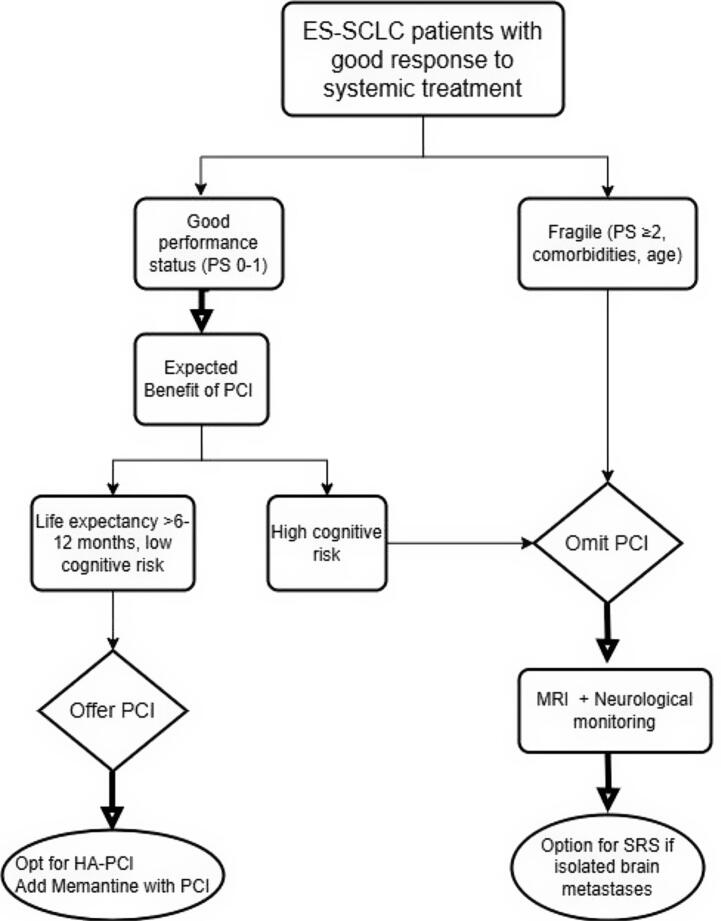
Decision-Making Approach for PCI in ES-SCLC. Abbreviations: ES-SCLC, extensive-stage small cell lung cancer; PS, performance status; PCI, prophylactic cranial irradiation, HA-PCI, hippocampal-avoidance PCI, MRI, magnetic resonance imaging, SRS, stereotactic radiosurgery;

The flowchart (Fig. 2) below illustrates the suggested possible approach to adopt.

### Awaiting future results

Given the current evidence, it is essential to follow the outcomes of ongoing trials such as MAVERICK (SWOG1827) and PRIMALung. Their objectives and endpoints have been intentionally aligned to enable a potential joint analysis offering a new perspective [47]. Both trials primarily aim to clarify the role of PCI in the immunotherapy era, while also exploring hippocampal-sparing strategies. Several factors will guide the future selection of ES-SCLC patients who could benefit from PCI. These include the patient's response to initial chemotherapy or immunotherapy, as those with a better response may have improved outcomes with PCI. Additionally, patient characteristics like age, performance status, and comorbidities, as well as the growing use of brain imaging surveillance (e.g., MRI), will help identify those who may not need PCI. The ASTRO guidelines, recommending a consultation with a radiation oncologist, favored decision-making based on personalized medicine rather solely on imaging such as surveillance with MRI.

Recent advances have significantly improved our understanding of the molecular basis of brain metastasis and may contribute to predicting outcomes of brain radiotherapy [4]. Initially, SCLC was considered a uniform disease arising from neuroendocrine cells, largely due to the frequent loss of tumor suppressor genes Rb1 and TP53, as well as its neuroendocrine/epithelial differentiation. However, further research revealed significant heterogeneity among SCLC tumors [48]. More recently, a molecular classification system emerged, focusing on the expression of key transcription factors in SCLC cells. This system identifies four subtypes: SCLC-A (ASCL1-dominant), SCLC-N (NEUROD1-dominant), SCLC-P (POU2F3-dominant), SCLC-I (low expression of all three factors, with an inflamed gene signature). SCLC-A may be more responsive to chemotherapy and radiotherapy while SCLC-I subtype is more responsive to PD-1/PD-L1 inhibitors [49].

Incorporating artificial intelligence (AI) into the decision-making process, by analyzing large datasets including the molecular profile of the tumor and predicting outcomes, could further enhance the precision of identifying patients who would benefit from PCI. As our understanding of ES-SCLC evolves, these factors, combined with AI-driven algorithms, will enable more personalized treatment decisions.

## Conclusion

In conclusion, the role of PCI in ES-SCLC remains a subject of ongoing debate, particularly in the context of the emerging use of immunotherapy. Historically, PCI has been shown to reduce brain metastases and improve survival in SCLC patients, particularly those with a positive response to chemotherapy. However, with the introduction of immunotherapy, which has demonstrated significant improvements in OS and intracranial PFS, the necessity of PCI has come into question. Studies have shown mixed results, with some suggesting PCI delays brain metastases without offering a significant survival benefit, while others point to the potential of MRI surveillance as an alternative. Given the advancements in treatment and the associated risks of PCI, including cognitive side effects and long-term complications, personalized approaches to treatment are crucial. Ongoing clinical trials like MAVERICK and PRIMALung are crucial to truly understand the role of PCI in ES-SCLC, particularly with the evaluation of hippocampal sparing.

## Funding statements

There was no external funding or financial support received for the research, development, or publication of this review.

## Clinical trial information

Not applicable.

## Data sharing statement

The data supporting the finding of this review are available from the corresponding author upon reasonable request.

## CRediT authorship contribution statement

The primary and author of the review: Dr Tala Najdi, Data Collection and Assembly: Dr. A.R, Dr. M.C and Dr T.Z, Tables Constructions: Dr. T. N and Dr. A.R, Manuscript Writing: Dr T. N, Dr A.R, Dr F.A, Dr C.H and Dr H. K, Final approval of manuscript: All authors.

## Declaration of Competing Interest

The authors declare that they have no known competing financial interests or personal relationships that could have appeared to influence the work reported in this paper.

## References

[1] Wang W.Z., Shulman A., Amann J.M., Carbone D.P., Tsichlis P.N. (2022 Nov). Small cell lung cancer: Subtypes and therapeutic implications. Semin Cancer Biol.

[2] Chen Y., Paz-Ares L., Reinmuth N., Garassino M.C., Statsenko G., Hochmair M.J. (2022 Jun). Impact of Brain Metastases on Treatment patterns and Outcomes with First-Line Durvalumab Plus Platinum-Etoposide in Extensive-Stage SCLC (CASPIAN): a Brief Report. JTO Clin Res Rep.

[3] Noronha V., Sekhar A., Patil V.M., Menon N., Joshi A., Kapoor A. (2020 Oct). Systemic therapy for limited stage small cell lung carcinoma. J Thorac Dis.

[4] Zhu Y, Cui Y, Zheng X, Zhao Y, Sun G. Small-cell lung cancer brain metastasis: From molecular mechanisms to diagnosis and treatment. Biochimica et Biophysica Acta (BBA) - Molecular Basis of Disease. 2022 Dec 1;1868(12):166557. 10.1016/j.bbadis.2022.166557 36162624

[5] Aupérin A., Arriagada R., Pignon J.P., Péchoux C.L., Gregor A., Stephens R.J. (1999 Aug 12). Prophylactic Cranial Irradiation for patients with Small-Cell Lung Cancer in complete Remission. N Engl J Med.

[6] Slotman B., Faivre-Finn C., Kramer G., Rankin E., Snee M., Hatton M. (2007 Aug 16). Prophylactic cranial irradiation in extensive small-cell lung cancer. N Engl J Med.

[7] Takahashi T., Yamanaka T., Seto T., Harada H., Nokihara H., Saka H. (2017 May). Prophylactic cranial irradiation versus observation in patients with extensive-disease small-cell lung cancer: a multicentre, randomised, open-label, phase 3 trial. Lancet Oncol.

[8] Ready N.E., Pang H.H., Gu L., Otterson G.A., Thomas S.P., Miller A.A. (2015 May 20). Chemotherapy with or without Maintenance Sunitinib for Untreated Extensive-Stage Small-Cell Lung Cancer: a Randomized, Double-blind, Placebo-Controlled phase II Study-CALGB 30504 (Alliance). J Clin Oncol.

[9] Keller A., Ghanta S., Rodríguez-López J.L., Patel A., Beriwal S. (2021 Nov). Utility of Prophylactic Cranial Irradiation for Extensive-Stage Small-Cell Lung Cancer in the MRI Screening Era. Clin Lung Cancer.

[10] Gaebe K., Erickson A.W., Li A.Y., Youssef A.N., Sharma B., Chan K.K.W. (2024 Jan). Re-examining prophylactic cranial irradiation in small cell lung cancer: a systematic review and meta-analysis. EClinicalMedicine.

[11] Gondi V., Paulus R., Bruner D.W., Meyers C.A., Gore E.M., Wolfson A. (2013 Jul 15). Decline in tested and self-reported cognitive functioning after prophylactic cranial irradiation for lung cancer: pooled secondary analysis of Radiation Therapy Oncology Group randomized trials 0212 and 0214. Int J Radiat Oncol Biol Phys.

[12] Gjyshi O., Ludmir E.B., Pezzi T.A., Boyce-Fappiano D., Dursteler A.E., Mitin T. (2019 Aug 14). Evolving Practice patterns in the use of Prophylactic Cranial Irradiation for Extensive-Stage Small Cell Lung Cancer. JAMA Netw Open.

[13] Kalemkerian GP, Loo BW, Akerley W, Attia A, Bassetti M, Boumber Y, et al. NCCN Guidelines Insights: Small Cell Lung Cancer, Version 2.2018. Journal of the National Comprehensive Cancer Network. 2018 Oct 1;16(10):1171–82. 10.6004/jnccn.2018.0079 30323087

[14] Dingemans A.M.C., Früh M., Ardizzoni A., Besse B., Faivre-Finn C., Hendriks L.E. (2021 Jul). Small-cell lung cancer: ESMO Clinical Practice guidelines for diagnosis, treatment and follow-up☆. Ann Oncol.

[15] Daly M.E., Ismaila N., Decker R.H., Higgins K., Owen D., Saxena A. (2021 Mar 10). Radiation Therapy for Small-Cell Lung Cancer: ASCO Guideline Endorsement of an ASTRO Guideline. JCO.

[16] Le Péchoux C., Dunant A., Senan S., Wolfson A., Quoix E., Faivre-Finn C. (2009 May). Standard-dose versus higher-dose prophylactic cranial irradiation (PCI) in patients with limited-stage small-cell lung cancer in complete remission after chemotherapy and thoracic radiotherapy (PCI 99-01, EORTC 22003-08004, RTOG 0212, and IFCT 99-01): a randomised clinical trial. Lancet Oncol.

[17] Wolfson A.H., Bae K., Komaki R., Meyers C., Movsas B., Le Pechoux C. (2011 Sep 1). Primary analysis of a phase II randomized trial Radiation Therapy Oncology Group (RTOG) 0212: impact of different total doses and schedules of prophylactic cranial irradiation on chronic neurotoxicity and quality of life for patients with limited-disease small-cell lung cancer. Int J Radiat Oncol Biol Phys.

[18] Yang D.X., Jairam V., Park H.S., Decker R.H., Chiang A.C., Gross C.P. (2020). Use of prophylactic cranial irradiation in patients with extensive-stage small cell lung cancer receiving immunotherapy. JCO.

[19] Liu S.V., Reck M., Mansfield A.S., Mok T., Scherpereel A., Reinmuth N. (2021 Feb 20). Updated overall Survival and PD-L1 Subgroup Analysis of patients with Extensive-Stage Small-Cell Lung Cancer Treated with Atezolizumab, Carboplatin, and Etoposide (IMpower133). JCO.

[20] Horn L., Mansfield A.S., Szczęsna A., Havel L., Krzakowski M., Hochmair M.J. (2018 Dec 6). First-Line Atezolizumab plus Chemotherapy in Extensive-Stage Small-Cell Lung Cancer. N Engl J Med.

[21] Higgins K.A., Curran W.J., Liu S.V., Yu W., Brockman M., Johnson A. (2020 Dec 1). Patterns of Disease Progression after Carboplatin/Etoposide + Atezolizumab in Extensive-Stage Small-Cell Lung Cancer (ES-SCLC). Int J Radiat Oncol Biol Phys.

[22] Paz-Ares L., Dvorkin M., Chen Y., Reinmuth N., Hotta K., Trukhin D. (2019 Nov 23). Durvalumab plus platinum–etoposide versus platinum–etoposide in first-line treatment of extensive-stage small-cell lung cancer (CASPIAN): a randomised, controlled, open-label, phase 3 trial. Lancet.

[23] Chen Y., Paz-Ares L.G., Dvorkin M., Trukhin D., Reinmuth N., Garassino M.C. (2020). First-line durvalumab plus platinum-etoposide in extensive-stage (ES)-SCLC (CASPIAN): Impact of brain metastases on treatment patterns and outcomes. JCO.

[24] Rudin C.M., Awad M.M., Navarro A., Gottfried M., Peters S., Csőszi T. (2020 Jul 20). Pembrolizumab or Placebo Plus Etoposide and platinum as First-Line Therapy for Extensive-Stage Small-Cell Lung Cancer: Randomized, Double-blind, phase III KEYNOTE-604 Study. J Clin Oncol.

[25] Owonikoko T.K., Park K., Govindan R., Ready N., Reck M., Peters S. (2021 Apr 20). Nivolumab and Ipilimumab as Maintenance Therapy in Extensive-Disease Small-Cell Lung Cancer: CheckMate 451. J Clin Oncol.

[26] Cheng Y., Fan Y., Zhao Y., Huang D., Li X., Zhang P. (2024 Jul). Tislelizumab Plus platinum and Etoposide Versus Placebo Plus platinum and Etoposide as First-Line Treatment for Extensive-Stage SCLC (RATIONALE-312): a Multicenter, Double-blind, Placebo-Controlled, Randomized, phase 3 Clinical Trial. J Thorac Oncol.

[27] Daumas A., Bigarre C., Boucekine M., Zaccariotto A., Kaeppelin B., Mogenet A. (2024 Dec 9). Lack of Prophylactic Cranial Irradiation for Extensive Small-Cell Lung Cancer in Real Life, with the Emergence of Immunotherapy. Cancers (Basel).

[28] Gross A.J., Sheikh S., Kharouta M., Chaung K., Choi S., Margevicius S. (2023 Dec). The Impact of Prophylactic Cranial Irradiation and Consolidative Thoracic Radiation Therapy for Extensive Stage Small-Cell Lung Cancer in the transition to the Chemo-Immunotherapy Era: a Single Institution Series. Clin Lung Cancer.

[29] SWOG Cancer Research Network. MRI Brain Surveillance Alone Versus MRI Surveillance and Prophylactic Cranial Irradiation (PCI): A Randomized Phase III Trial in Small-Cell Lung Cancer (MAVERICK) [Internet]. clinicaltrials.gov; 2024 May [cited 2025 Mar 1]. Report No.: NCT04155034. Available from: https://clinicaltrials.gov/study/NCT04155034.

[30] Levy A., Berghmans T., Koller M., Fournier B., Mauer M., Andratschke N. (2024 Dec). PRIMALung (EORTC-1901): PRophylactic cerebral irradiation (PCI) or active brain MAgnetic resonance imaging (MRI) surveillance in small-cell lung cancer (SCLC) patients. Lung Cancer.

[31] Wang L. Real-world Study of Prophylactic Cranial Irradiation After Immunotherapy Combined With Chemotherapy for Extensive-stage Small Cell Lung Cancer [Internet]. clinicaltrials.gov; 2021 Sep [cited 2025 Mar 1]. Report No.: NCT04947774. Available from: https://clinicaltrials.gov/study/NCT04947774.

[32] Lone A.H., Salunkhe R., Sugumar V., Zhan L.J., Ye X.Y., Bezjak A. (2025 Mar). Real-world outcomes of prophylactic cranial irradiation utilization and efficacy for patients with extensive-stage small cell lung cancer treated with consolidative thoracic radiotherapy. Clin Transl Radiat Oncol.

[33] Zhou Z. A Prospective Randomized Controlled Study for Prophylactic Cranial Irradiation After Chemotherapy and Thoracic Radiation Therapy in Patients With Extensive-stage Small Cell Lung Cancer [Internet]. clinicaltrials.gov; 2020 Aug [cited 2025 Mar 1]. Report No.: NCT04535739. Available from: https://clinicaltrials.gov/study/NCT04535739.

[34] Bruni A., Scotti V., Zerella M.A., Bertolini F., Imbrescia J., Olmetto E. (2024 Nov). Current Radiotherapy Management of Extensive-Stage Small-Cell Lung Cancer in the Immunotherapy Era: an Italian National Survey on Behalf of the Italian Association of Radiotherapy and Clinical Oncology (AIRO). Curr Oncol.

[35] Wen P., Wang T.F., Li M., Yu Y., Zhou Y.L., Wu C.L. (2020 Feb 1). Meta-analysis of prophylactic cranial irradiation or not in treatment of extensive-stage small-cell lung cancer: the dilemma remains. Cancer/radiothérapie.

[36] Rusthoven C.G., Yamamoto M., Bernhardt D., Smith D.E., Gao D., Serizawa T. (2020 Jul). Evaluation of First-line Radiosurgery vs Whole-Brain Radiotherapy for Small Cell Lung Cancer Brain Metastases. JAMA Oncol.

[37] Aizer AA, Tanguturi SK, Shi DD, Catalano PJ, Shin KY, Ricca I, et al. Stereotactic Radiosurgery in Patients With Small Cell Lung Cancer and 1-10 Brain Metastases: A Multi-Institutional, Phase II, Prospective Clinical Trial. J Clin Oncol. 2025 Jul 11;JCO2500056. 10.1200/JCO-25-00056 40644657

[38] Kim H., Keller A., Beriwal S., Smith K.J., Vargo J.A. (2021 Dec 1). Cost-Effectiveness of Prophylactic Cranial Irradiation Versus MRI Surveillance for Extensive-Stage Small Cell Lung Cancer. Int J Radiat Oncol Biol Phys.

[39] Sun A., Bae K., Gore E.M., Movsas B., Wong S.J., Meyers C.A. (2011 Jan 20). Phase III trial of prophylactic cranial irradiation compared with observation in patients with locally advanced non-small-cell lung cancer: neurocognitive and quality-of-life analysis. J Clin Oncol.

[40] Gondi V., Pugh S.L., Tome W.A., Caine C., Corn B., Kanner A. (2014 Dec 1). Preservation of memory with conformal avoidance of the hippocampal neural stem-cell compartment during whole-brain radiotherapy for brain metastases (RTOG 0933): a phase II multi-institutional trial. J Clin Oncol.

[41] Rodríguez de Dios N., Couñago F., Murcia-Mejía M., Rico-Oses M., Calvo-Crespo P., Samper P. (2021 Oct 1). Randomized phase III Trial of Prophylactic Cranial Irradiation with or without Hippocampal Avoidance for Small-Cell Lung Cancer (PREMER): a GICOR-GOECP-SEOR Study. J Clin Oncol.

[42] Belderbos J.S.A., De Ruysscher D.K.M., De Jaeger K., Koppe F., Lambrecht M.L.F., Lievens Y.N. (2021 May). Phase 3 Randomized Trial of Prophylactic Cranial Irradiation with or without Hippocampus Avoidance in SCLC (NCT01780675). J Thorac Oncol.

[43] Zeng H, Schagen SB, Hendriks LEL, Sánchez-Benavides G, Jaspers JPM, Manero RM, et al. Impact of HA-PCI on self-reported cognitive functioning and brain metastases in small-cell lung cancer: Pooled findings of NCT01780675 and PREMER trials. Lung Cancer [Internet]. 2025 Jan 1 [cited 2025 Sep 16];199. Available from: https://www.lungcancerjournal.info/article/S0169-5002(24)00570-1/fulltext. 10.1016/j.lungcan.2024.108036 39615412

[44] (2024). ResearchGate [internet].

[45] Brown P.D., Gondi V., Pugh S., Tome W.A., Wefel J.S., Armstrong T.S. (2020 Apr 1). Hippocampal Avoidance during Whole-Brain Radiotherapy Plus Memantine for patients with Brain Metastases: phase III Trial NRG Oncology CC001. J Clin Oncol.

[46] Brown P.D., Pugh S., Laack N.N., Wefel J.S., Khuntia D., Meyers C. (2013 Oct). Memantine for the prevention of cognitive dysfunction in patients receiving whole-brain radiotherapy: a randomized, double-blind, placebo-controlled trial. Neuro Oncol.

[47] Levy A., Rusthoven C.G., Brown P.D., Le Péchoux C., Faivre-Finn C. (2025 Mar). Prophylactic Cranial Irradiation for patients with SCLC-A New Perspective in the Immunotherapy Era. J Thorac Oncol.

[48] Liang J., Guan X., Bao G., Yao Y., Zhong X. (2022 Nov). Molecular subtyping of small cell lung cancer. Semin Cancer Biol.

[49] Gay C.M., Allison Stewart C., Park E.M., Diao L., Groves S.M., Heeke S. (2021 Mar 8). Patterns of transcription factor programs and immune pathway activation define four major subtypes of SCLC with distinct therapeutic vulnerabilities. Cancer Cell.

