# Supplementary material for: Prophylactic cranial irradiation in ES-SCLC: The ongoing debate from the past to the immunotherapy Era”
Source: Clin Transl Radiat Oncol. 2026 Mar 21;58:101153. doi: 10.1016/j.ctro.2026.101153 (PMC13049582; doi:10.1016/j.ctro.2026.101153)
Supplement: Supplementary Data 1 [file mmc1.docx]

Supplementary Table on Generalizability of Study Population(s)

| **Condition** | **Description** |
| --- | --- |
| Disease, problem, or condition under investigation | Prophylactic cranial irradiation (PCI) in extensive-stage small cell lung cancer (ES-SCLC) |
| Relevant considerations of disease, problem, or condition in relation to: | *Note any relevant considerations in boxes below:* |
| Sex and gender | The studies included in the review represent both male and female patients, with a predominance of male participants reflecting disease prevalence. No significant sex-based limitations were identified. |
| Age | The review includes data from a broad age range. Findings are generally applicable across age groups. |
| Race or ethnic group | The reviewed literature includes diverse population, across various racial and ethnic groups. |
| Geography |  |
| Other considerations | \|  \| \| --- \|  \| The review captures the evolution of PCI use across different eras, including traditional and immunotherapy-integrated treatment settings making it relevant to the current practice \| \| --- \| |
| **Study** | **Review** |
| Overall assessment of generalizability of the study population | The review synthesizes data from diverse clinical settings and populations, supporting the general applicability. |
